# Abnormal Spontaneous Neural Activity in Parkinson’s Disease With “pure” Apathy

**DOI:** 10.3389/fnins.2020.00830

**Published:** 2020-08-04

**Authors:** Hai-Hua Sun, Jian-Bin Hu, Jing Chen, Xue-Yang Wang, Xiao-Li Wang, Ping-Lei Pan, Chun-Feng Liu

**Affiliations:** ^1^Department of Neurology, Affiliated Yancheng Hospital, School of Medicine, Southeast University, Yancheng, China; ^2^Department of Radiology, Affiliated Yancheng Hospital, School of Medicine, Southeast University, Yancheng, China; ^3^Department of Neurology and Suzhou Clinical Research Center of Neurological Disease, The Second Affiliated Hospital of Soochow University, Suzhou, China; ^4^Department of Emergency, Affiliated Yancheng Hospital, School of Medicine, Southeast University, Yancheng, China; ^5^Institute of Neuroscience, Soochow University, Suzhou, China

**Keywords:** apathy, Parkinson’s disease, amplitude of low-frequency fluctuation, nucleus accumbens, dorsal anterior cingulate cortex

## Abstract

**Background:**

Apathy is one of the most common non-motor symptoms of Parkinson’s disease (PD). However, its pathophysiology remains unclear.

**Methods:**

We analyzed resting-state functional magnetic resonance imaging (MRI) data acquired at a 3.0T MRI scanner using the amplitude of low-frequency fluctuation (ALFF) metric in 20 *de novo*, drug-naïve, non-demented PD patients with apathy (PD-A), 26 PD patients without apathy (PD-NA) without comorbidity of depressive or anxious symptoms, and 23 matched healthy control (HC) subjects.

**Results:**

We found that the ALFF decreased significantly in the bilateral nucleus accumbens, dorsal anterior cingulate cortex (ACC), and left dorsolateral prefrontal cortex in patients with PD-A compared to patients with PD-NA and HC subjects. Furthermore, apathy severity was negatively correlated with the ALFF in the bilateral nucleus accumbens and dorsal ACC in the pooled patients with PD.

**Conclusion:**

The present study characterized the functional pattern of changes in spontaneous neural activity in patients with PD-A. With the aim to better elucidate the pathophysiological mechanisms responsible for these changes, this study controlled for the potentially confounding effects of dopaminergic medication, depression, anxiety, and global cognitive impairment. The findings of the current study add to the literature by highlighting potential abnormalities in mesocorticolimbic pathways involved in the development of apathy in PD.

## Introduction

Apathy is one of the most common non-motor symptoms of Parkinson’s disease (PD), with a prevalence of almost 40% (Den Brok et al., 2015). Apathy is defined as a syndrome of diminished motivation (reduced goal-directed behavior, goal-directed cognitive activity, and emotional expression) that persists over time (Robert et al., 2009). Apathy can precede the onset of motor symptoms in PD and is associated with poor outcomes (Pont-Sunyer et al., 2015). As such, it is crucial that apathy is recognized early to allow for timely intervention. However, the pathophysiology of apathy in PD remains unclear, although it has been hypothesized that disruptions of the basal ganglia circuits of the prefrontal cortex are heavily involved (Levy and Dubois, 2006; Pagonabarraga et al., 2015). The degeneration of dopaminergic and serotonergic pathways has been proposed as a possible pathophysiological mechanism for apathy in PD (Maillet et al., 2016).

Resting-state functional magnetic resonance imaging (rs-fMRI) is a non-invasive neuroimaging technique, based on blood oxygen level dependent (BOLD) signals, that has gained much attention over the past decade. The amplitude of low-frequency fluctuations (ALFF) is a promising and reliable metric that can be used to measure local spontaneous brain activity via rs-fMRI (Yang et al., 2007). This metric has been employed frequently in cases of PD and other brain disorders, and it has been suggested that abnormalities in the ALFF may serve as potential imaging markers for some disorders, such as amnestic mild cognitive impairment, schizophrenia, and major depressive disorder (Pan et al., 2017; Wang et al., 2017; Gong et al., 2020). A recent meta-analysis of 15 rs-fMRI studies showed that a decreased ALFF in the putamen of PD patients was the most consistent finding (Ding et al., 2018). This contributes to the characterization of the underlying neurophysiology of PD. However, to date, only two studies have reported on apathy-related changes in the ALFF that are associated with PD (Skidmore et al., 2013; Shen et al., 2018). A previous study showed that apathy was associated with a decreased ALFF in the left supplementary motor cortex and an increased ALFF in the right orbitofrontal cortex and the right middle frontal cortex (Skidmore et al., 2013). Another study showed a decreased ALFF in the left orbital middle frontal gyrus and the bilateral superior frontal gyrus of PD patients with apathy, relative to those without apathy. Additionally, it was found that a lower ALFF in the right superior frontal gyrus was correlated with higher apathy scores (Shen et al., 2018). However, the aforementioned rs-fMRI studies may have been subject to confounding factors, such as anti-PD or anti-apathy medications. This may reduce the utility of their findings. Previous studies have demonstrated dopaminergic modulation of resting-state functional activity and connectivity in PD (Wu et al., 2009; Baik et al., 2014; Berman et al., 2016; Gao et al., 2017; Zhong et al., 2019). In addition, depression (Skidmore et al., 2013; Wen et al., 2013; Luo et al., 2014; Hu et al., 2015; Zhi et al., 2019) anxiety (Skidmore et al., 2013; Wang X. et al., 2018) and cognitive impairment (Wang Z. et al., 2018) were associated with ALFF abnormalities in PD. Distinct and separable ALFF patterns that were correlated with apathy, depression, and motor symptoms were observed in the same PD sample (Skidmore et al., 2013).

To control for potential confounding factors, the present study examined the ALFF in drug-naïve, non-demented PD patients without comorbidity of depression and anxiety to characterize the neuropathological mechanisms of “pure” apathy. We hypothesized that apathy in PD would be associated with ALFF abnormalities in the basal ganglia circuits of the prefrontal cortex.

## Materials and Methods

### Study Participants

A total of 69 subjects, including 26 newly diagnosed drug-naïve PD patients without apathy (PD-NA), 20 newly diagnosed drug-naïve PD patients with apathy (PD-A), and 23 matched healthy control (HC) subjects were recruited for the current study between December 2017 and November 2018. The clinical diagnoses of all idiopathic PD patients were established according to the consensus of two experienced neurologists, specializing in movement disorders, using the United Kingdom Parkinson’s disease brain bank criteria (Hughes et al., 1992). Additionally, all patients attended a follow-up, within 12 months of diagnosis, for diagnostic re-evaluation and treatment of parkinsonian symptoms. The study was approved by the local ethics committee. All participants gave written informed consent in accordance with the Declaration of Helsinki.

Enrolled patients were assessed with a detailed series of neurological examinations, a brief neuropsychiatric assessment, and structural MRI evaluations of the brain by two specialists, experienced in assessing movement disorders. All subjects were assessed with the following measures: (1) the Mini Mental State Examination (MMSE) (Katzman et al., 1988) and Montreal Cognitive Assessment (MoCA) (Nasreddine et al., 2005) to assess global cognitive function; (2) the Hamilton Anxiety Rating Scale (HAMA) (Hamilton, 1960) and Hamilton Depression Rating Scale (HAMD-17) (Hamilton, 1960) to evaluate the severity of anxiety and depressive symptoms, respectively; and (3) the frontal assessment battery (FAB) (Dubois et al., 2000) trail making test-B (TMT-B) (Arbuthnott and Frank, 2000) and interference task portion of the Stroop test (IT-ST) (Barbarotto et al., 1998) to measure frontal/executive functions. The Unified Parkinson’s Disease Rating Scale, part III (UPDRS-III) (Goetz et al., 2007) and the Hoehn and Yahr (HY) scale (Hoehn and Yahr, 1967) were utilized to evaluate motor disability and the stage of PD, respectively. The Apathy Scale (AS) (Starkstein et al., 1992) a popular measure of apathy in PD, which includes 14 items and a score ranging from 0 to 42—was used to categorize PD patients into the apathic (AS score ≥ 14) and non-apathic (AS score < 14) groups.

To be included in the study (i.e., fulfill the inclusion criteria), patients had to (1) fit the diagnostic criteria for idiopathic PD (Hughes et al., 1992) (2) be older than 45 years of age, (3) be a newly diagnosed patient who had never taken medication for Parkinsonism before the fMRI scan. Patients were re-evaluated to confirm the diagnosis of PD after one year of enrollment, and (4) have no depressive or anxiety disorders (HAMD-17 score < 7 and HAMA score < 7, respectively), which were evaluated by a trained psychiatrist, and 5) have no dementia. The optimal cut-off points of MMSE score for dementia screening were 16/17 for illiterate, 19/20 for individuals with 1–6 years of education, and 23/24 for individuals with 7 or more years of education (Li et al., 2016). The optimal cut-off points of MoCA score were 13/14 for illiterate individuals, 19/20 for individuals with 1 to 6 years of education, and 24/25 for individuals with 7 or more years of education (Lu et al., 2011). Exclusion criteria for patients included (1) a diagnosis of a secondary or atypical parkinsonism; (2) a psychiatric diagnosis, a history of head trauma, alcoholism, drug dependence, or drug abuse; (3) cerebral lesions identified by CT or MRI scan; and (4) a moderate to severe head tremor, sufficient to yield movement artifacts during neuroimaging acquisitions. Age, gender, and handedness-matched HC individuals were recruited from the local community. All HCs had medical histories that were free of neurologic or psychiatric diseases. All the data were collected on the day of the MRI scan.

### Imaging Data Acquisition

All rs-fMRI data were obtained at our hospital using a 3.0T MRI scanner (GE Discovery MR 750, Milwaukee, WI, United States) with an eight-channel head coil. Participants were instructed to rest but to remain awake, keep their eyes closed, keep their heads still, and avoid focusing their thoughts on a specific subject during fMRI scanning. The rs-fMRI data of each subject were collected using a the gradient-echo planar imaging (GRE-EPI) sequence with the following parameters: repetition time = 2000 ms, echo time = 30 ms, flip angle = 90°, matrix = 64 × 64, field of view = 240 × 240, 35 continuous axial slices covering the entire brain with a slice thickness = 4 mm, inter-slice space = 0 mm, NEX = 1, voxel size = 3.75 mm × 3.75 mm × 4 mm, and time points = 230, producing a total of 8050 images.

### Image Processing

All the rs-fMRI data were processed using Data Processing and Analysis for (Resting-State) Brain Imaging (DPABI), version 4.2 (Yan et al., 2016) a user-friendly pipeline analysis toolkit based on Statistical Parametric Mapping (SPM12)^1^ running on MATLAB, version R2013b (MathWorks, MA, United States). Analytical processes of ALFF were described in detail elsewhere (Yan et al., 2016). Here, the procedures are briefly summarized in the following pipeline including the check of the image quality from each participant; conversion of EPI DICOM images to the NIFTI format, removal of the first 10 functional volumes; slice-timing correction; realignment; head-motion correction (Friston-24 head motion parameters, head motion < 2.0 mm translation and < 2° rotation, as well as 0.2 mm in mean frame-wise displacement); regression of white matter (WM), cerebrospinal fluid (CSF), and global mean signal; spatial normalization by diffeomorphic anatomical registration through exponentiated lie algebra (DARTEL) to the standard Montreal Neurological Institute (MNI) brain space with a resampling voxel size of 3 × 3 × 3 mm^3^; smoothness with a full width at half maximum Gaussian kernel of 4 mm^3^; and filtration with a routine temporal band-pass (0.01–0.1 Hz) after linear detrending.

Following the preprocessing stage, the BOLD time course of each voxel was converted to the frequency domain via a fast Fourier transform, to produce the power spectrum. The square root of the power spectrum was then computed and further averaged within a specific frequency domain (0.01–0.1 Hz). The resulting averaged square root at each voxel within the whole-brain mask represented the ALFF, reflecting absolute strength or intensity of spontaneous neural activity. In order to reduce the global effects of variability, the ALFF of each voxel was divided by the global mean ALFF, to standardize these values prior to further statistical analysis.

### Statistics

Differences in age, education level, MMSE score, MOCA score, AS score, HAMD-17 score, HAMA score, FAB score, TMT-B score, and IT-ST score between the three groups were assessed using one-way analyses of variance (ANOVA), while differences according to the distribution of sexes were evaluated with a chi-squared test. Independent two-sample *t*-tests were conducted to compare illness durations, UPDRS-III scores, and HY stage between the two patient groups using Bonferroni correction. Possible relationships between AS score and demographic, clinical, and neuropsychological measures in pooled PD patients were assessed using a correlational analysis (Pearson’s correlation). The significance level was defined as *P* < 0.05. The demographic, clinical, and neuropsychological data above were analyzed using IBM SPSS software, version 23.0 (SPSS, Inc., Chicago, IL, United States).

Voxel-wise comparisons of the ALFF maps were conducted among the three groups, using an ANCOVA model adjusted for age, gender, education level, MMSE score, MoCA score, HAMD-17 score, HAMA score, FAB score, TMT-B score, IT-ST score, and FD. Voxel-wise post-hoc analysis was further performed for pair-wise group comparisons. For the post-hoc analysis, we utilized a mask generated from the brain areas that showed significant ALFF differences among the three groups. Statistical maps were thresholded at *P* < 0.05 with family wise error (FWE) corrected using a permutation-based approach with threshold-free cluster enhancement (TFCE), implemented within DPABI (Chen et al., 2018). We also performed two-sample *t*-tests of each pair using a region of interest (ROI) approach by extracting the mean ALFF *z*-values from the brain areas that showed significant ALFF differences among the three groups. Bonferroni correction (*P* < 0.05) was used to determine significance. To further examine potential core regions that could account for apathy, Pearson correlation analyses were conducted to investigate the relationships between the mean *z*-values in the ROIs and the AS scores. These analyses controlled for demographic, clinical, and neuropsychological variables, which were significantly correlated with AS scores in pooled PD patients at *P* < 0.05 with Bonferroni correction.

## Results

### Demographic and Clinical Characteristics

Table 1 summarizes the demographic, clinical, and neuropsychological characteristics of the 26 patients with PD-NA (14 men, mean age 59.61 ± 9.97 years), 20 patients with PD-A (14 men, mean age 59.85 ± 8.92 years), and 23 HC subjects (13 men, mean age 59.47 ± 10.79 years). The three groups had significantly different scores on the AS (F = 131.98, *p* < 0.001), TMT-B (F = 14.82, *p* < 0.001), IT-ST (F = 24.78, *p* < 0.001) and FAB (F = 29.37, *p* < 0.001). There were no significant differences in age (F = 0.008, *p* = 0.99), gender (F = 0.66, *p* = 52), education level (F = 0.17, *p* = 0.84), handedness (F = 0.00, *p* = 1.00), MMSE score (F = 0.31, *p* = 0.74), MOCA score (F = 0.22, *p* = 0.80), HAMD-17 score (F = 0.098, *p* = 0.91), or HAMA score (F = 0.14, *p* = 0.87) between the groups. No significant differences were observed according to illness duration (*p* = 0.78), UPDRS-III score (*p* = 0.35), or HY stage (*p* = 0.82), between the two PD groups. In the pooled PD patients, AS scores were significantly correlated with TMT-B scores (*r* = 0.7, *p* < 0.001) and IT-ST scores (*r* = 0.81, *p* < 0.001), whereas AS scores were not significantly correlated with other demographic, clinical, or neuropsychological measures.

**TABLE 1:** Demographic and clinical features for the participants.

Characteristics	PD-A (*n* = 20)	PD-NA (*n* = 26)	HC (*n* = 23)	Statistic	Post-hoc two sample *T* test		
					PD-A vs. HC	PD-NA vs. HC	PD-A vs. PD-NA
Age	59.85 ± 8.92	59.61 ± 9.97	59.47 ± 10.79	F = 0.008, *P* = 0.99	*P* = 0.90	*P* = 0.96	*P* = 0.94
Sex (M/F)	14/6	14/12	13/10	F = 0.66, *P* = 0.52	*P* = 0.38	*P* = 0.85	*P* = 0.28
Education (years)	7.05 ± 5.53	7.12 ± 5.13	7.91 ± 5.95	F = 0.17, *P* = 0.84	*P* = 0.90	*P* = 0.82	*P* = 0.94
Handness (R/L)	20/0	26/0	23/0	F = 0.00, *P* = 1.00	*P* = 1.00	*P* = 1.00	*P* = 1.00
PD duration (years)	2.05 ± 0.81	1.98 ± 0.87	—	T = 0.28	—	—	*P* = 0.78
HY	1.90 ± 0.60	1.94 ± 0.62	—	T = 0.23	—	—	*P* = 0.82
UPDRS-III	18.05 ± 3.03	17.08 ± 3.74	—	T = 0.95	—	—	*P* = 0.35
MMSE	24.95 ± 3.27	25.88 ± 4.25	25.57 ± 4.37	F = 0.31, *P* = 0.74	*P* = 0.62	*P* = 0.78	*P* = 0.44
MoCA	22.90 ± 3.99	23.96 ± 5.36	23.61 ± 6.33	F = 0.22, *P* = 0.80	*P* = 0.67	*P* = 0.82	*P* = 0.51
AS	19.95 ± 3.35	8.04 ± 2.65	7.35 ± 2.57	F = 131.98, *P* < 0.001	*P* < 0.001	*P* = 0.40	*P* < 0.001
HAMD-17	2.90 ± 1.12	2.73 ± 1.40	2.87 ± 1.63	F = 0.098, *P* = 0.91	*P* = 0.94	*P* = 0.73	*P* = 0.69
HAMA	2.70 ± 1.22	2.62 ± 1.44	2.48 ± 1.53	F = 0.14, *P* = 0.87	*P* = 0.61	*P* = 0.74	*P* = 0.84
FAB	13.65 ± 1.79	15.77 ± 1.39	17.04 ± 1.19	F = 29.37, *P* < 0.001	*P* < 0.001	*P* = 0.003	*P* < 0.001
TMT-B (seconds)	189.70 ± 28.09	168.19 ± 27.33	146.13 ± 23.03	F = 14.82, *P* < 0.001	*P* < 0.001	*P* = 0.005	*P* = 0.007
IT-ST	13.50 ± 2.16	16.92 ± 4.09	19.96 ± 1.99	F = 24.78, *P* < 0.001	*P* < 0.001	*P* = 0.005	*P* = 0.002

*PD-A, Parkinson’s disease with apathy; PD-NA, Parkinson’s disease without apathy; HC, healthy control; M, male; F, female; R, right; L, left; HY, Hoehn and Yahr; UPDRS-III, Unified Parkinson’s Disease Rating Scale, part III; MMSE, Mini Mental State Examination; MoCA, Montreal Cognitive Assessment; AS, Apathy Scale; HAMD, Hamilton Depression Rating Scale; HAMA, Hamilton Anxiety Rating Scale; FAB, frontal assessment battery; TMT-B, trail making test part B; IT-ST, Interference task of Stroop test.*

### ALFF Analysis

**FIGURE 1:**
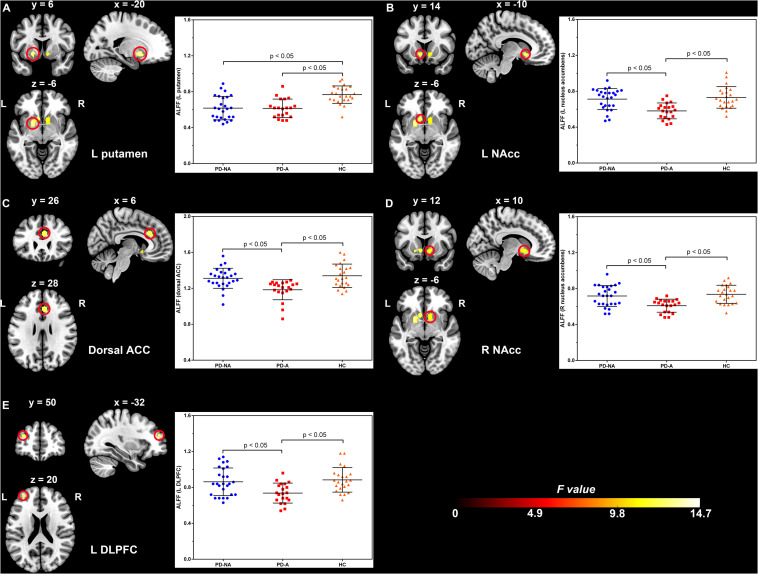
Significantly different clusters and their ALFF values among the three groups (*P* < 0.05, FWE corrected). **(A)**, left putamen; **(B)**, left nucleus accumbens; **(C)**, dorsal anterior cingulate cortex; **(D)**, right nucleus accumbens; **(E)**, left dorsolateral prefrontal cortex; ALFF, amplitude of low-frequency fluctuation; FWE, family wise error; PD-NA, Parkinson’s disease without apathy; PD-A, Parkinson’s disease with apathy; HC, healthy control; L, left; R, right; NAcc, nucleus accumbens; ACC, anterior cingulate cortex; DLPFC, dorsolateral prefrontal cortex. The bar graphs represent the clusters where the mean ALFF was significantly different between each pair of the three groups and the error bar. Blue dots, red squares, and yellow triangles represent the distributions of ALFF values extracted from the brain regions in the PD-NA, PD-A, and HC groups, respectively. Differences were identified by region of interest analyses.

**FIGURE 2:**
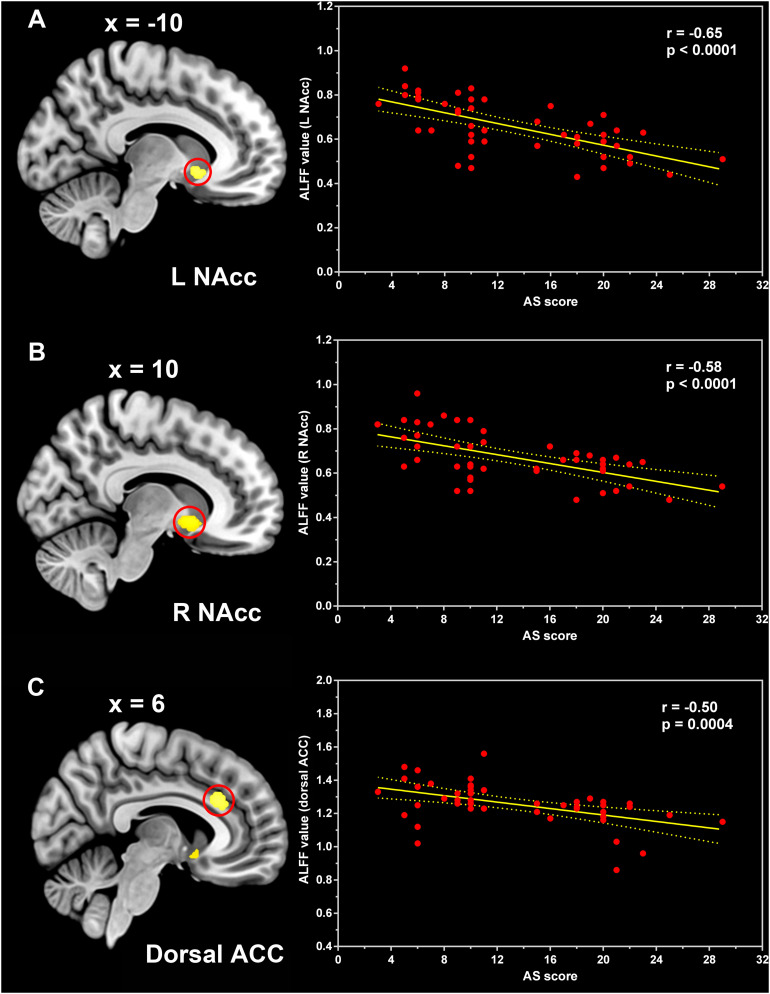
Correlation between the ALFF and AS scores. Pearson correlation analyses between the ALFF values in the left nucleus accumbens **(A)**, right nucleus accumbens **(B)**, and dorsal anterior cingulate cortex **(C)** and AS scores of the pooled PD patients. ALFF, amplitude of low-frequency fluctuation; NAcc, nucleus accumbens; ACC, anterior cingulate cortex; AS, Apathy Scale.

The one-way ANCOVA of ALFF maps among the three groups (FWE with TFCE correction *P* < 0.05) revealed significant differences in the left putamen (Figure 1A), left nucleus accumbens (Figure 1B), dorsal anterior cingulate cortex (ACC, Figure 1C), right nucleus accumbens (Figure 1D), and left dorsolateral prefrontal cortex (DLPFC, Figure 1E). These results are shown in Table 2. Voxel-wise post-hoc pair-wise group comparisons (Table 3) showed that lower ALFF values in left nucleus accumbens, dorsal ACC, right nucleus accumbens, and left DLPFC in the PD-A group relative to the PD-NA group. Compared to the HC group, the PD-NA group had a lower ALFF in the left putamen. Relative to HC subjects, PD-A patients had a lower ALFF in the left putamen, left nucleus accumbens, dorsal ACC, right nucleus accumbens, and left DLPFC. ROI analyses (Table 2) further validated above results. Correlation analyses showed that AS scores in the pooled PD sample were negatively correlated with the ALFF in the left nucleus accumbens (Figure 2A, *r* = −0.65, *p* < 0.0001), right nucleus accumbens (Figure 2B, *r* = −0.58, *p* < 0.0001), and dorsal ACC (*r* = −0.50, *p* = 0.0004).

**TABLE 2:** ANCOVA and ROI analyses of ALFF differences among the three groups.

Brain regions	Peak MNI (x, y, z)	Cluster size (mm^3^)	Max F value	ALFF value (Mean with SD)			ROI contrast results
				PD-A	PD-NA	HC	
L putamen	−20, 6, −6	150	14.70	0.61 (0.10)	0.62 (0.13)	0.77 (0.10)	PD-A < HC, PD-NA < HC, PD-NA = PD-A
L nucleus accumbens	−10, 14, −6	71	12.36	0.58 (0.09)	0.71 (0.12)	0.73 (0.12)	PD-A < PD-NA, PD-A < HC, PD-NA = HC
Dorsal ACC	6, 26, 28	102	11.89	1.18 (0.11)	1.31 (0.11)	1.34 (0.13)	PD-A < PD-NA, PD-A < HC, PD-NA = HC
R nucleus accumbens	10, 12, −6	95	10.65	0.61 (0.07)	0.72 (0.12)	0.74 (0.10)	PD-A < PD-NA, PD-A < HC, PD-NA = HC
L DLPFC	−32, 50, 20	59	11.24	0.74 (0.11)	0.86 (0.15)	0.88 (0.14)	PD-A < PD-NA, PD-A < HC, PD-NA = HC

*ANCOVA, analysis of covariance; ROI, region of interest; ALFF, amplitude of low-frequency fluctuation; MNI, Montreal Neurological Institute; SD, standard deviations; PD-A, Parkinson’s disease with apathy; PD-NA, Parkinson’s disease without apathy; HC, healthy control; L, left; ACC, anterior cingulate cortex; R, right; DLPFC, dorsolateral prefrontal cortex.*

**TABLE 3:** *Post-hoc* pairwise comparisons of ALFF differences among the three groups using voxel-wise analyses.

Brain regions	Peak MNI coordinates (x, y, z)	Cluster size (mm^3^)	Max *T* value
**PD-A < HC**			
L putamen	−22, 8, −2	108	−5.78
L nucleus accumbens	−10, 12, −4	54	−5.25
Dorsal ACC	8, 24, 24	124	−5.53
R nucleus accumbens	8, 10, −8	101	−4.37
L DLPFC	−36, 54, 16	68	−4.65
**PD-NA < HC**			
L putamen	−19, 10, −6	97	−5.36
**PD-A < PD-NA**			
L nucleus accumbens	−8, 10, −6	82	−6.94
Dorsal ACC	6, 28, 30	96	−5.32
R nucleus accumbens	10, 14, −4	78	−4.24
L DLPFC	−32, 44, 26	47	−4.09

*ALFF, amplitude of low-frequency fluctuation; MNI, Montreal Neurological Institute; PD-A, Parkinson’s disease with apathy; PD-NA, Parkinson’s disease without apathy; HC, healthy control; L, left; ACC, anterior cingulate cortex; R, right; DLPFC, dorsolateral prefrontal cortex.*

## Discussion

Using the ALFF approach, via rs-fMRI, we demonstrated that altered spontaneous, regional neural activity is associated with apathy in drug-naïve PD patients with “pure” apathy (i.e., without comorbidity of dementia, depression, and anxiety). We found significantly decreased ALFF values in the bilateral nucleus accumbens, dorsal ACC, and left DLFPC in patients with PD-A compared to patients with PD-NA and HC subjects. Moreover, the apathy level of PD patients was negatively correlated with the ALFF in the nucleus accumbens and dorsal ACC. These findings add to the literature suggesting that abnormalities in mesocorticolimbic pathways lead to apathy in PD (Pagonabarraga et al., 2015). To the best of our knowledge, the current ALFF study is the first to control for the potentially confounding effects of dopaminergic medication, depression, anxiety, and global cognitive impairment in PD patients with apathy. As such, this research may better elucidate the pathophysiological mechanisms underlying apathy.

The nucleus accumbens, a part of the ventral striatum (also called the limbic striatum), is a critical node of the mesocorticolimbic system that is implicated in reward processing and as playing a key role in normal motivated behavior (Le Heron et al., 2018). It has been reported that dopaminergic dysfunction within the ventral tegmental area–nucleus accumbens pathway predicts apathetic behavior in the animal model of PD (1-methyl-4-phenyl-1,2,3,6-tetrahydropyridine [MPTP]-lesioned monkeys) (Brown et al., 2012). In addition to evidence that dopaminergic denervation underlies apathy in PD (Pagonabarraga et al., 2015) have suggested that β-amyloidopathy (Zhou et al., 2020) and serotonergic degeneration (Maillet et al., 2016) play prominent roles in the nucleus accumbens. Additionally, reductions in functional connectivity, predominantly involving the limbic striatal and frontal areas, have been observed in apathetic PD patients. Further, the severity of apathy was negatively correlated with functional connectivity in these circuits (Baggio et al., 2015). Structural neuroimaging research has also shown that apathy in PD is associated with atrophy in the nucleus accumbens (Carriere et al., 2014; Martinez-Horta et al., 2017). Furthermore, hypometabolism in the ventral striatum was found to be a risk factor for becoming apathetic after subthalamic nucleus deep brain stimulation in patients with PD without depression or dementia (Robert et al., 2014). Using rs-fMRI, the present study documented decreased neural activity in the nucleus accumbens in apathic PD patients relative to non-apathetic patients. Moreover, our study showed negative correlations between neural activity in this region and the apathy level of PD patients. These findings provide further evidence that dysfunction in the nucleus accumbens contributes to the development of apathy in PD and highlight the importance of this dysfunction.

Notably, the present study also found decreased ALFF magnitudes in the dorsal ACC of patients with PD-A relative to patients with PD-NA and HC subjects. The dorsal ACC forms the frontostriatal circuits, in combination with the ventral striatum, and is implicated as a critical region for normal motivated behavior (Le Heron et al., 2018). A wealth of neuroimaging evidence shows that disruption of the components or connectivity of these circuits is strongly linked to apathy across brain disorders, which suggests that common brain systems subserve apathy across different pathologies (Le Heron et al., 2018). In addition, apathy in PD has been shown to be associated with greater levels of atrophy in the dorsal ACC (Alzahrani et al., 2016). The present study showed that apathy severity was negatively correlated with ALFF magnitudes in the dorsal ACC. This provides further evidence that the occurrence of apathy is associated with local brain dysfunction in this region.

We found a decreased ALFF in the left DLPFC of patients with PD-A relative to those with PD-NA and HC subjects. The DLPFC is an important region for executive control (Seeley et al., 2007). The DLPFC–caudate circuits have been implicated as playing a key role in all forms of apathy (Pagonabarraga et al., 2015). Our study revealed that apathy severity was positively correlated with executive dysfunction, which is consistent with previous reports (Femiano et al., 2018; Brown et al., 2019). However, no correlation was observed between apathy severity and the ALFF in this region, after controlling for executive function, in the present study. The absence of this association may indicate that executive deficits associated with apathy do not fully explain the clinical correlates and the underlying mechanisms of apathy in PD (Martinez-Horta et al., 2013, 2017).

Our study did not replicate a previous case-control report on ALFF alterations in apathy in PD (Shen et al., 2018). This discrepancy could be due to differences in age, duration of illness, severity of motor disability, and severity of depressive and anxious symptoms in PD patients between the two studies. Medication status (whether taking antiparkinsonian drugs or not) may also account for this discrepancy. Previous studies have shown that local neural activity could be modified by dopaminergic medication (Kwak et al., 2012; Xu et al., 2019). Our study controlled for a number of potential confounding factors, such as the effects of medication and comorbidity of depressive and anxious symptoms in PD. As such, it may better elucidate the neurobiological basis of apathy associated with alterations in spontaneous, regional neural activity.

Several limitations to this preliminary study should be considered. One limitation of the present study is the relatively small sample size. Additional investigations with larger samples are required to validate our findings. The AS is a validated measure for evaluating the severity of apathy in PD (Starkstein et al., 1992). However, apathy is a multidimensional construct, involving cognitive, behavioral, and emotional symptoms, and the AS has been recognized as having limitations in subtyping these dimensions of apathy (Lopez et al., 2019). Toward understanding the mechanisms behind the multidimensional nature of apathy, future rs-fMRI studies should use more refined and comprehensive tools to detect the substructure of apathy, such as the Dimensional Apathy Scale, (Radakovic and Abrahams, 2018). Our study only investigated the relationship between apathy and deficits of global cognitive and frontal/executive functions in patients with PD. More detailed evaluations of other cognitive functions (memory, language, praxia, processing speed/attention/working memory, and visuospatial abilities) would give more insights regarding their relationship with apathy (D’iorio et al., 2018). Our study mainly focused on apathy related regional neural activity alterations controlling for dementia, depression, anxiety and medication in PD. However, PD is a prevalent and highly heterogeneous disorder with many motor and non-motor subtypes. Future work that characterizes the separate neural substrates of different phenotypes of PD is encouraged. Measurement of the ALFF is a reliable rs-fMRI analytical method that is widely used for characterizing local neural activity and may provide targets for interventions (Ding et al., 2018). This method can be complemented by analyses of network or functional connectivity to provide more information on the neural mechanisms of apathy, in future studies.

## Conclusion

In summary, our data illustrated that apathy in drug naïve PD patients was associated with decreased intrinsic neural activity in the bilateral nucleus accumbens, dorsal ACC, and left DLFPC, after controlling for dementia, depression, and anxiety. These results add to the literature suggesting that the dysfunction of mesocorticolimbic pathways is involved in the underlying pathophysiology of apathy in PD.

## Data Availability Statement

The raw data supporting the conclusions of this article will be made available by the authors, without undue reservation.

## Ethics Statement

The studies involving human participants were reviewed and approved by Ethics Committee of Affiliated Yancheng Hospital, School of Medicine, Southeast University. The patients/participants provided their written informed consent to participate in this study.

## Author Contributions

H-HS and C-FL designed the study. H-HS, P-LP, J-BH, JC, X-LW, and X-YW analyzed and interpreted the data. H-HS and P-LP drafted the manuscript. JC and C-FL performed critical revision of the manuscript. All authors approved the final version for submission.

## Conflict of Interest

The authors declare that the research was conducted in the absence of any commercial or financial relationships that could be construed as a potential conflict of interest.

## Footnotes

1 http://www.fil.ion.ucl.ac.uk/spm/software/spm12/
